# Identification of Deleterious Mutations in *Myostatin* Gene of Rohu Carp (*Labeo rohita*) Using Modeling and Molecular Dynamic Simulation Approaches

**DOI:** 10.1155/2016/7562368

**Published:** 2016-02-25

**Authors:** Kiran Dashrath Rasal, Vemulawada Chakrapani, Swagat Kumar Patra, Shibani D. Mohapatra, Swapnarani Nayak, Sasmita Jena, Jitendra Kumar Sundaray, Pallipuram Jayasankar, Hirak Kumar Barman

**Affiliations:** Fish Genetics and Biotechnology Division, Central Institute of Freshwater Aquaculture (ICAR), Bhubaneswar, Odisha 751 002, India

## Abstract

The myostatin (MSTN) is a known negative growth regulator of skeletal muscle. The mutated myostatin showed a double-muscular phenotype having a positive significance for the farmed animals. Consequently, adequate information is not available in the teleosts, including farmed rohu carp,* Labeo rohita*. In the absence of experimental evidence, computational algorithms were utilized in predicting the impact of point mutation of rohu myostatin, especially its structural and functional relationships. The four mutations were generated at different positions (p.D76A, p.Q204P, p.C312Y, and p.D313A) of MSTN protein of rohu. The impacts of each mutant were analyzed using SIFT, I-Mutant 2.0, PANTHER, and PROVEAN, wherein two substitutions (p.D76A and p.Q204P) were predicted as deleterious. The comparative structural analysis of each mutant protein with the native was explored using 3D modeling as well as molecular-dynamic simulation techniques. The simulation showed altered dynamic behaviors concerning RMSD and RMSF, for either p.D76A or p.Q204P substitution, when compared with the native counterpart. Interestingly, incorporated two mutations imposed a significant negative impact on protein structure and stability. The present study provided the first-hand information in identifying possible amino acids, where mutations could be incorporated into MSTN gene of rohu carp including other carps for undertaking further* in vivo* studies.

## 1. Introduction

The myostatin, belonging to the member of the Transforming Growth Factor-*β* (TGF*β*) superfamily, was initially identified in mice by McPherron et al. [1]. It is a negative regulator of skeletal muscle growth in mammalian species [2–5]. It inhibits myoblast specification and differentiation via downregulating* Pax3*,* Myf-5,* and* MyoD* expressions in myoblasts [6]. The mice carrying a targeted dominant mutation in* MSTN* gene resulted in the increased skeletal muscle mass due to the combined effects of muscle hypertrophy and hyperplasia [7, 8]. Mice lacking the biologically active region of the MSTN C-terminal developed muscular hypertrophy in the chest and buttock [1]. Cattle (*Bos taurus*) is the first species in which a mutation in the* MSTN* gene showed the double-muscling phenotype. Subsequently, similar growth effects were reported in dogs, cattle, buffalo, porcine, and sheep [9–14]. In mammals, the desired effects were obtained due to mutation, positioned in either p.D76A, p.Q204A, p.C312Y, or p.C313Y. Therefore, the mutation mediated improvement of the skeletal muscle mass provided an avenue for improving growth related production traits in the farmed animals [15]. A transgenic mouse, containing a p.D76A mutation (within the leader peptide) in the* MSTN* gene, significantly increased muscle mass [16]. The experimentally incorporated mutations positioned at p.C313Y in cattle and p.D76A in mice as well as dog led to dramatic skeletal muscle growth.

The teleosts,* MSTN* gene, is well-conserved throughout evolution, where two distinct* MSTN* gene isoforms such as MSTNa and MSTNb were found in some fish species [17–20]. In zebrafish (*Danio rerio*), the roles of* MSTN* gene concerning muscle development and growth were envisaged by inhibiting their function using its prodomain [16, 21, 22]. The* MSTN* gene characterization as well as knock-out and indel experiments in some fish species such that of yellow catfish [23], medaka [24], and rainbow trout [25] showed double-muscling phenotype with hyperplasia and hypertrophy.

Thus, it would be of interest to identify each mutation in* MSTN* gene that disturbs its function, leading to improved muscle growth in commercially important aquaculture species. Commercial applications require in-depth analyses in the diversified teleosts. However, limited information is available whether the fish with mutated MSTN could display a similar phenotype or not. The Indian major carp,* Labeo rohita* (popularly known as rohu), is an economically important freshwater fish in India as well as other Asian countries [26–28]. The sequence data of rohu, catla (*Catla catla*), and zebrafish (*Danio rerio*) myostatin genes are available in the public domain (GenBank Acc. numbers HQ850573, HQ850575, and AY323521). However, its physiological functions in Indian major carps including rohu are lacking. In this study, we intended to assess the impact of p.D76A, p.Q204A, p.C312Y, and p.C313Y mutations in* MSTN* gene of rohu carp.

Direct experimental studies linked to incorporating mutation in a particular gene of interests have been laborious and time-consuming. The computational study can efficiently produce useful information to rationalize and guide in undertaking further experimental studies [28–30]. These point mutations affect gene expressions and their products by altering change in their functional activities [31]. The computational tools such as SIFT, PolyPhen, I-Mutant 2.0, PANTHER, and PROVEAN are more useful for the pin-pointing impact analysis of point mutation linking the gene function or gene products [29, 32–37]. Consequently, the combination of a different algorithm will improve the accuracy of results or predicted effects of particular mutation [38]. SNPs with amino acid (aa) substitutions can disturb protein folding and its stability, leading to altered protein function and protein-protein interactions including its expression [39–42].

We have used several computational tools for determining the impact of the selected point mutation on* MSTN* gene. To get insight into the atomic-level changes and the dynamic behavior of the molecule on to the particular mutations, we have used molecular dynamic simulation approaches. We have identified the potential mutations, proposed modeled structure of the mutant proteins, and compared them with the native protein. The present* in silico* study is helpful for categorizing precise mutation in MSTN gene for the purpose of selecting a definite gene targeting site.

## 2. Materials and Methods

### 2.1. Retrieval of Data

The MSTN protein sequence of rohu,* Labeo rohita,* was retrieved from Uniprot with ID C9DE96_LABRO (http://www.uniprot.org/uniprot/C9DE96). SMART (a Simple Modular Architecture Research Tool) was used for identification and annotation including architectures of the mobile domain in the MSTN (http://smart.embl-heidelberg.de/).

### 2.2. Prediction of SNP Effects in MSTN Protein Using Sequence-Based Tools

For elucidation of the impact of SNPs in MSTN protein of rohu, several* in silico* tools comprising of SIFT, PANTHER, PROVEAN, and I-Mutant 2.0 were utilized. The SIFT (Sorting Intolerant from Tolerant) algorithm is useful for determining the impact of single amino acid substitution in the resultant protein (http://sift.jcvi.org/). The tolerance index, that is, score of SIFT, in the tune of ≤0.05, determined the nsSNPs (nonsynonymous SNPs) on resultant protein [43]. The higher the tolerance index, the less likely the impact on AASs. Subsequently, PANTHER (Protein Analysis through Evolutionary Relationships) was used so as to validate the predicted output from SIFT analysis. The PANTHER cSNP tool that estimates the likelihood of a particular nonsynonymous (amino acid changing) coding SNP to cause a functional impact on the protein (http://www.pantherdb.org/tools/csnp). It calculates the subPSEC score based on alignments of evolutionarily related proteins [44]. The score more than −3 is predicted as a less deleterious while less than −3 is predicted as deleterious. We have used this tool for detecting the impact of AASs at selected positions in the MSTN protein.

Further, PROVEAN and I-Mutant 2.0 algorithm were used for assessing the impacts of SNPs in MSTN protein of rohu. PROVEAN (Protein Variation Effect Analyzer) is a tool which predicts impact of an amino acid substitution or indel on the biological functions of a particular protein (http://provean.jcvi.org/index.php). This algorithm allows for the best-balanced separation between the deleterious and neutral AASs, based on a threshold value. A query sequence was provided in FASTA format and score for prediction was based on default threshold score −2.5. The score <−2.5 indicates that variant is deleterious and >−2.5 is considered as a neutral [45]. I-Mutant 2.0 is a Support Vector Machine- (SVM-) based web server for the automatic prediction of protein stability changes upon single-site mutations (http://folding.biofold.org/i-mutant/i-mutant2.0.html). The predicted free energy change (DDG) is based on the differential unfolding Gibbs free energy change (kcal/Mol) between mutant and native proteins [33].

### 2.3. MSTN Protein Structure Prediction

We have searched homologous 3D structure for MSTN protein of rohu in the RCSB PDB data bank. Due to the absence of homologous 3D structure, we have used I-TASSER (Iterative Threading ASSEmbly Refinement) server, which is an online platform for predicting protein structure and function (http://zhanglab.ccmb.med.umich.edu/I-TASSER/). It is based on multiple threading alignments and iterative structural assembly simulations. The prediction of the accuracy of the model depends upon a confidence score (C-score) determined by the quality of the threading alignments and structural assembly refinement simulations. In this server, we have submitted query sequence (FASTA format) of MSTN protein for obtaining 3D model.

The best 3D model was selected according to rank based on C-score, TM-score, and RMSD value. The 3D structure of MSTN was visualized by PyMOL (http://www.pymol.org/) and UCSF-CHIMERA (https://www.cgl.ucsf.edu/chimera/). The PyMoL is an open source of molecular visualization tools, for interactive visualization and analysis of molecular structures. The superimposition of both MSTN native and mutant models was carried out by using UCSF-CHIMERA (https://www.cgl.ucsf.edu/chimera/). The SwissPDBViewer was used for analyzing RMS deviation by superimposing both native and mutant structures (http://spdbv.vital-it.ch/).

The structural evaluation and stereochemical quality assessments for both native and mutant models were carried out by using the SAVES (Structural Analysis and Verification Server), which is an integrated server (http://nihserver.mbi.ucla.edu/SAVES/). The ProSA (Protein Structure Analysis) web server (https://prosa.services.came.sbg.ac.at/prosa.php) was used for refinement and validation of protein structures [46]. The ProSA was used for checking model structural quality with potential errors, where program shows a plot of its residue energies and *Z*-scores to determine the overall quality of the model. The protein folding rate was calculated using PRORATE (prediction of protein folding rates) server (http://sunflower.kuicr.kyoto-u.ac.jp/~sjn/folding/).

### 2.4. Investigation of SNP Effects in MSTN 3D Protein by Structure-Based Tools

The mutant model was generated using PyMoL tool. The H-bonding patterns were studied using the PyMoL visualization tool. Both native and mutant 3D structures of MSTN were used for molecular dynamic (MD) simulations.

The MD simulation was performed using the program of GROMACS 4.6.5 package (Groingen Machine for Chemical Simulations) running on Ubuntu Linux platform 14.04. Respective mutant and native models were solvated independently within a dodecahedron box with SPC216 (single point charge 216) water molecules at a marginal radius of 10 Å. The energy minimization was carried out for 5000 iterations/steps with a tolerance of 100 kJ/mol by steepest descent algorithm (implementing OPLS force field). Emtol convergence criterion was set up to 1000 kcal/Mol. The coulomb interactions were truncated at 1.0 nm. The ionization states of the residues were set to a pH 7.0. Finally, MD simulation for 50000 steps (10 ns) was carried out. The resulted trajectory files were analyzed by using gromacs packages so as to differentiate RMSD and RMSF values between native and mutant structures of MSTN. The graphs were constructed using XMGrace on Linux platform itself.

## 3. Results

### 3.1. Mutation at Either p.D76A or p.Q204P of Rohu MSTN Protein Is Deleterious

Several computational tools were applied to investigate the structural and functional impacts of targeted single nucleotide polymorphisms (SNPs) present in the coding region of the* MSTN* gene of rohu (*L. rohita*). The dataset of possible nsSNPs was selected based upon previously reported observations in the mammalian system. In this study, we have used both sequence-based (SIFT, PANTHER, PROVEAN, and I-Mutant) and structure-based (I-TASSER) algorithms. We have generated mutations by p.D76A, p.Q204P, p.C312Y, and p.D313A amino acid (aa) substitutions in the rohu MSTN protein using the PyMoL tool (Table 1). The most of the algorithms are based upon evolutionary principle. Subsequently, we have analyzed the effect of each mutation using a series of computational tools. Initially, SIFT, PANTHER, PROVEAN, and I-Mutant 2.0 analyses predicted p.D76A and p.Q204P substitutions as the most deleterious with score 0.02 and 0.04, respectively (SIFT score ≤ 0.05). Contrary to this, p.C312Y and p.D313A mutations generated quite variable results, when analyzed with different algorithms of SIFT and PANTHER tools. The resultant significant differential outputs with SIFT and PANTHER algorithms could be due to consideration of different protein sequence alignments used to characterize the variants; the same was also documented earlier [47]. The functional impacts of aa substitutions (AAAs) were further validated by providing FASTA sequence of the protein as a query along with a change in residue(s) to PROVEAN, which is capable of classifying impacts of AASs or indels on functional protein [45]. Except p.D313A, the remaining three substitutions were classified as deleterious. To improve prediction accuracy, I-Mutant 2.0 algorithm was applied. This I-Mutant 2.0 also categorized p.C312Y with increased stability, while others were classified as deleterious substitutions.

### 3.2. Structure-Based Analyses Also Supported the Deleterious Nature of p.D76A and p.Q204A Substitutions

**Figure 1:**
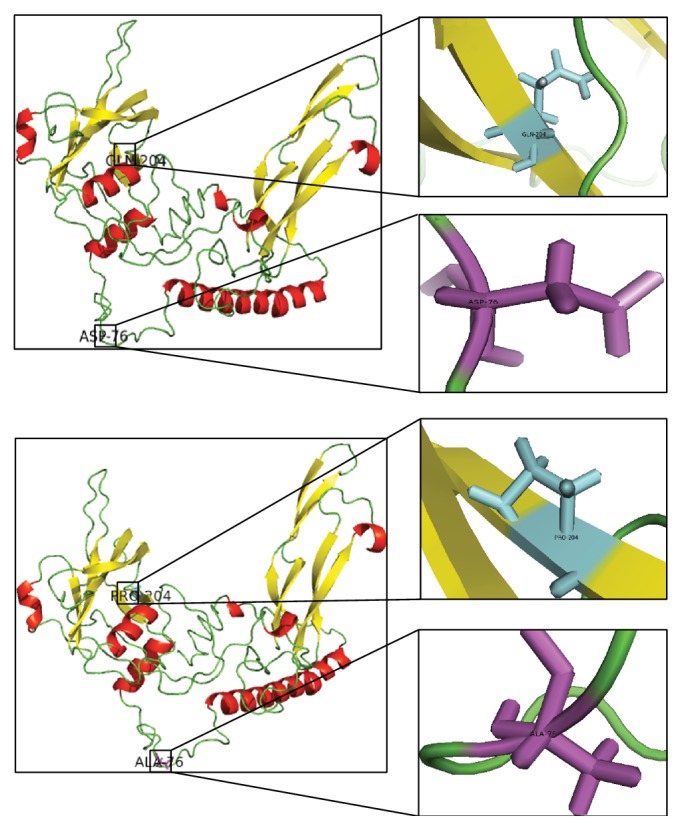
Predicted MSTN models by I-TASSER (both native and mutant) with mutated native and mutated residues.

The structural information could play a vital role in unraveling the molecular mechanism as well as being helpful for predicting the effects of nsSNPs on overall protein structure [48]. The modeling of rohu MSTN protein was performed by I-TASSER, which is a structure-based automated prediction algorithm. Ten templates were considered for modeling MSTN protein. The best 3D structure with a high confidence score (C-score) was selected and used for further investigations. The top model, bearing estimated C-score of 0.46, TM-score of 0.65 ± 0.13, and an RMSD value of 7.7 ± 4.3 Å, was finally selected (Figure 1).

**Figure 2:**
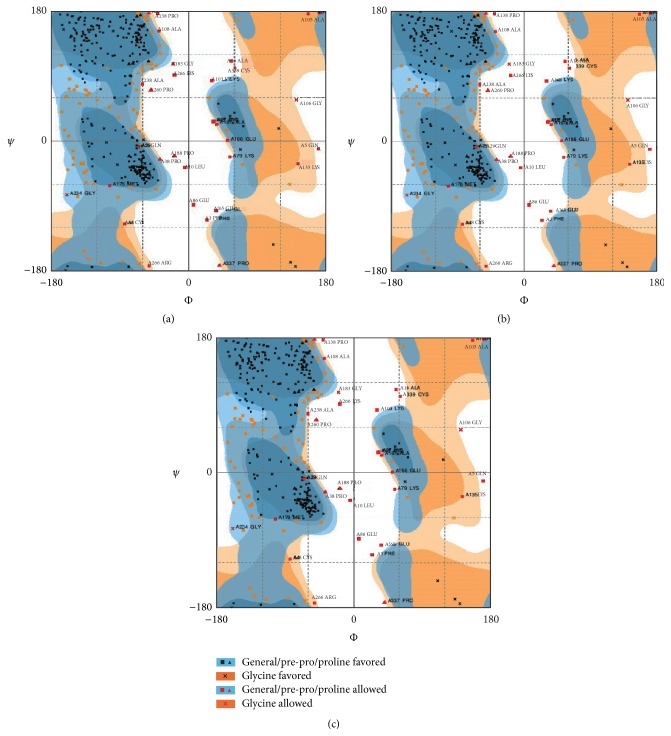
Ramachandran plot of MSTN mutant protein models (a, b, and c). The most favored regions, additional allowed regions, generously allowed regions, and disallowed regions are indicated as dark blue, light blue, light yellow, and white, respectively.

**Figure 3:**
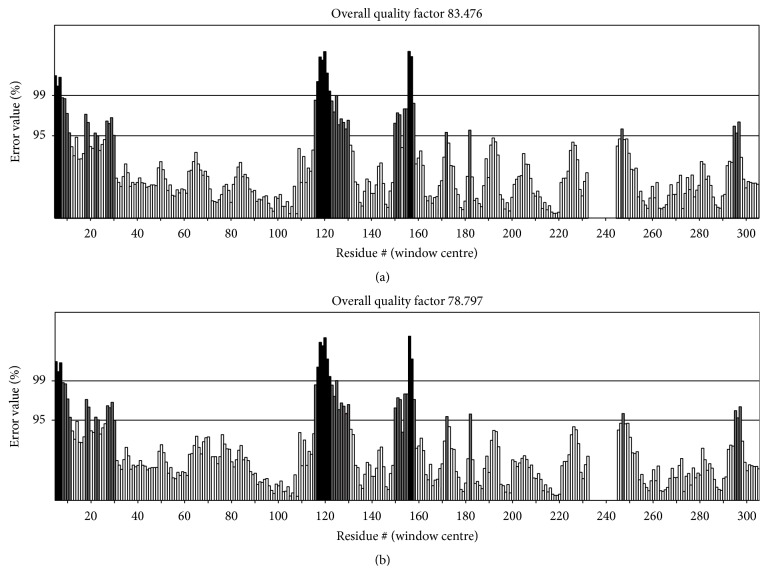
ERRAT plots for (a) MSTN native protein and (b) MSTN mutant protein model generated by SAVES. The plots for both proteins show overall quality factors 83.476 and 78.797, respectively. The grey region indicates error region 95% and 99%, white bars show region of lower error rate for protein folding, and black bars show misfolded region located distantly from active site. The below 95% value (i.e., rejection limit) indicates low resolution structure, while more than or 95% value shows high resolution structure.

The differential Ramachandran plot is depicted in Figure 2. In the plot, native protein of phi/psi angles for 73.1% residues was in the additional allowed regions with 18.3% in the most favored regions, while being 8.6% in disallowed regions. The comparative analysis between native and mutant structure was carried out, which revealed that 72.8%, 18.3%, and 8.9% residues lie in the most favored regions, additionally allowed regions, and disallowed regions, respectively. These results indicated that incorporated mutation enforced shifting of residues towards the disallowed region from the most favored regions in the case of p.Q204A mutation. No change was observed in p.D76A and native form of MSTN protein. The lowered ERRAT score for mutant in the tune of 78.797 as compared to native structure (83.476) also signified about the overall deteriorating quality of mutant MSTN (Figure 3). The ProSA analysis showed slightly deviated *Z*-score value in mutant protein (p.D76A and p.Q204A) as compared to native one. The score for native was −4.41, while mutants p.D76A and p.Q204A were −4.36 and 4.43, respectively. Together, these results provided the clue regarding possible conformational alterations for both mutant proteins (p.D76A and p.Q204A). The analysis of protein folding rate also specified that p.Q204A mutant and native possessed −18.38321, whereas folding rate varied with mutant p.D76A (−18.410992). It can be inferred from the structural analysis that the two deleterious mutations (p.D76A and p.Q204A) had fetched a severe change in the MSTN protein, and it is likely to disturb the protein function.

### 3.3. Analysis of Simulation at the Atomic Level Predicted the Deviated Functioning of Mutated (p.D76A or p.Q204A) MSTN

**Figure 4:**
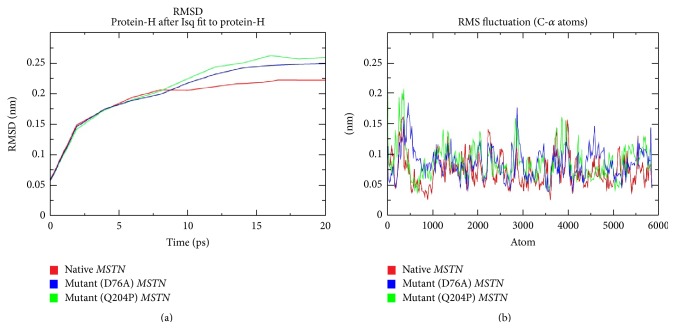
The RMSD and RMSF of both native and mutant MSTN structures of rohu carp (red color: native; blue: mutant MSTN (p.D76A); and green: mutant (p.Q204P).

In order to gain atomic-level insights as well as understanding of dynamic behavioral changes in mutant proteins, we performed MD simulations for both the native and mutant MSTNs. The MD simulation has been known to support the predictions given by sequence-based* in silico* tools. For this purpose, the RMSD of the protein backbone as a mean for functional time was examined. The RMSD for all protein-H atoms from the initial structure was reexamined to study the convergence of the protein system (Figure 4(a)). The mutant RMSD trajectory detected an abrupt rise of RMSD value starting from 5 ps onwards. The RMSD graph showed higher deviation in MSTN mutant (p.D76A and p.Q204P) structures as compared to the native counterpart (Figure 4(a)). To assess the effect of mutation on the MSTN structure, we also computed the RMSF values. The graphs show higher RMS fluctuations in MSTN mutant (both p.D76A and p.Q204P) structures as compared to the native structure (Figure 4(b)). Superimposition of native and mutant structures detected the RMS deviations in the tune of 0.049 Å, 0.029 Å, 0.030 Å, and 0.024 Å, respectively, for p.D76A, p.Q204P, p.C312Y, and p.D313A. The varied potential energies between native and both forms of mutants were also documented. The p.D76A mutant possessed −1.1378342*e* + 06 kJ/Mol and p.Q204P mutant contained −1.1424335*e* + 06 kJ/Mol (p.Q204P), whereas it possessed comparatively lesser potential energy in the tune of −1.170186*e* + 06 kJ/Mol for native counterpart. Further, the calculated total energies after MD simulated production run were −7.86233*e* + 05 kJ/Mol, −7.89900*e* + 05 kJ/Mol, and −8.4884*e* + 05 kJ/Mol, respectively, for p.D76A mutant, p.Q204P mutant, and the native structures. Thus, the differentially heightened energy requirements for both the mutant structures as compared to native one were suggestive of affecting (disturbed) the proper functioning of mutant MSTNs.

## 4. Discussion

Several evidences are available that the inhibition of MSTN gene led to dramatic growth rate/double-muscling phenotype in mammals. Alongside, attempts have been made to attenuate MSTN activities in fish muscle using mutagenesis approach. But, those approaches in fishes led to conflicting outcomes, possibly due to duplicated gene events or faulty selection of mutation sites. Also, the MSTN protein is extremely well-conserved between fish and other vertebrates. Nearly 90% of the amino acids (aa) are identical between fish and mammalian MSTN sequences. The experimental evidence in zebrafish [22] and medaka [49] demonstrated the increased number of skeletal muscle fibers via inhibition of MSTN. Remarkably, inhibition of MSTN could not lead to elevated muscle weight in both cases. Therefore, extensive studies are essential so as to understand the roles being played by MSTN in farmed fishes. The impact analysis as a consequence of mutation at a precise location is very important in order to gain economic benefits from farmed fishes.

Computational biology has long been a guiding force for undertaking experimental investigations. In the present work, we have generated mutations by amino acid substitutions at four positions such as p.D76A or p.Q204P or p.C312Y or p.D313A. SIFT, PANTHER, PROVEAN, and I-Mutant 2.0 based analyses predicted p.D76A and p.Q204P substitutions as the most deleterious. Those computational methods can predict (based on evolutionary principles) the effect of nsSNPs in the coding region of the genome [35, 50–52]. The 3D structure of a protein has been very helpful in predicting the effects of nsSNPs on protein structure-function [43, 48]. We have generated 3D structure of MSTN and validated it by using Ramachandran plot. Further, through MD simulation, we have compared RMSD values and total energy values between native and mutant structures. Based on estimated RMSD values, the computed energy provided the comparative information regarding structural stability and the deviation between the two (native and each mutant) structures. The deviated RMSD could have an impact on the functional activity and stability of protein [53]. The rise in RMSD values in the graph indicated that there was an enormous deviation between the native and mutant structures, pointing towards alterations in the structural and functional stabilities. We thus inferred that these nsSNPs could affect the stability of MSTN structure of rohu. Thus, it is likely that either p.D76A or p.Q204P substitution was deleterious in nature with regard to MSTN gene function. Our findings are partly in line with earlier experimental findings that a transgenic mouse, bearing p.D76A MSTN gene mutation, showed significantly improved muscle mass [16]. Also, mutations in the position p.Q204X, producing a premature stop codon in the N-terminal domain, resulted into double-muscling phenotype in Charolais and Limousine cattle breeds [54].

Thus, we could find that point mutation in the MSTN gene disturbs the protein structure and affects its function. Our findings also suggested that mutations in the N-terminal motif of TFGB chain could more effectively inhibit the MSTN gene activity. Our findings will provide a clue that improvement in muscular mass could be experimentally possible by incorporating p.D76A and/or p.Q204P mutations in rohu MSTN gene.

As the transcriptional regulation of MSTN genes is mediated by different myogenic factors, the previous works demonstrated that MSTN could able to negatively autoregulate its expression through a Smad7-dependent mechanism [55].

In recent times, gene targeting in predetermined position in the genome was made possible using technologies of zinc-finger nucleases (ZFNs), transcription activator-like effector nucleases (TALENs), and CRISPR/Cas9 in wide ranges of cell lines [56–63]. The site-specific nick by nucleases can then be repaired by error-prone nonhomologous end joining (NHEJ) resulting in animals carrying deletions or insertions at the cut site. It is also possible to disrupt any gene of interests by enforcing homologous recombination (HR) mediated repair at a particular nick site. Thus, it is possible to generate model fishes by creating indels and/or gene disruptions in the myostatin gene. This in turn will lead not only to uncover physiological roles of the myostatin gene but also to improve skeletal muscle growth in Indian farmed carps.

## 5. Conclusions

Our computational study identified that p.D76A or p.Q204P amino acid substitutions (nsSNPs) are deleterious for the structural and functional aspects of the rohu (*L. Rohita*) MSTN protein. We investigated the impact of the four variants on* MSTN* in the form of energy calculation and evolutionarily conserved residues. The SIFT, PANTHER, PROVEAN, and I-Mutant algorithms predicted decrease in protein stability. The MD simulation also confirmed that change in the dynamic behavior of mutant protein at the atomic level to the native counterpart. Based on the documented differences in total energy for native and mutant models, we speculated that the proper functioning of native MSTN protein would be disturbed.Our results suggested that these mutants in TGFb domain have a potential functional impact on MSTN genes in teleosts. The generated data from this study will be resourceful for undertaking future* in vivo* investigations so as to improve the growtgh rate of famed carps.

## Figures and Tables

**Table 1:** List of nsSNPs showing deleterious/nondeleterious scores as predicted by SIFT, PROVEAN, I-Mutant 2, and PANTHER.

Amino acids change Protein position	subPSEC score	*P*_deleterious_	Prediction by PANTHER-cSNP	PROVEAN score	PROVEAN prediction (cutoff = −2.5)	DDG value	I-Mutant stability prediction	SIFT score	Prediction
D76A	−2.63640	0.41009	Deleterious	−2.887	Deleterious	−0.75	Decreased	0.02	Deleterious
Q204P	−4.03755	0.73838	Tolerated	−2.942	Deleterious	−0.97	Decreased	0.04	Deleterious
C312Y	−11.6194	0.99982	Deleterious	−9.330	Deleterious	0.87	Increased	0.06	Tolerated
D313A	−1.72365	0.3933	Tolerated	−5.925	Neutral	0.92	Increased	0.09	Tolerated

## Abbreviations

MSTN: Myostatin
SIFT: Sorting Intolerant from Tolerant
SNP: Single nucleotide polymorphism
RMSD: Root means square deviations
PDB: Protein data bank
RMSF: Root means square fluctuations
MD: Molecular dynamics.
